# Expected affine: A registration method for damaged section in serial sections electron microscopy

**DOI:** 10.3389/fninf.2022.944050

**Published:** 2022-09-02

**Authors:** Tong Xin, Lijun Shen, Linlin Li, Xi Chen, Hua Han

**Affiliations:** ^1^Institute of Automation, Chinese Academy of Sciences, Beijing, China; ^2^School of Artificial Intelligence, University of Chinese Academy of Sciences, Beijing, China; ^3^The Center for Excellence in Brain Science and Intelligence Technology, Chinese Academy of Sciences, Shanghai, China; ^4^National Laboratory of Pattern Recognition, Institute of Automation, China Academy of Sciences, Beijing, China; ^5^School of Future Technology, University of Chinese Academy of Sciences, Beijing, China

**Keywords:** image registration, SSEM, broken sections, section fold, section crack

## Abstract

Registration is essential for the volume reconstruction of biological tissues using serial section electron microscope (ssEM) images. However, due to environmental disturbance in section preparation, damage in long serial sections is inevitable. It is difficult to register the damaged sections with the common serial section registration method, creating significant challenges in subsequent neuron tracking and reconstruction. This paper proposes a general registration method that can be used to register damaged sections. This method first extracts the key points and descriptors of the sections to be registered and matches them *via* a mutual nearest neighbor matcher. K-means and Random Sample Consensus (RANSAC) are used to cluster the key points and approximate the local affine matrices of those clusters. Then, K-nearest neighbor (KNN) is used to estimate the probability density of each cluster and calculate the expected affine matrix for each coordinate point. In clustering and probability density calculations, instead of the Euclidean distance, the path distance is used to measure the correlation between sampling points. The experimental results on real test images show that this method solves the problem of registering damaged sections and contributes to the 3D reconstruction of electronic microscopic images of biological tissues. The code of this paper is available at https://github.com/TongXin-CASIA/Excepted_Affine.

## Introduction

In connectomics studies, volume reconstruction reconstructs the neurite circuit from electron microscope images. The commonly used methods for obtaining electron microscope images of biological tissue include serial section electron microscopy (ssEM), focused ion beam scanning electron microscopy (FIB-SEM), and serial block-face scanning electron microscopy (SBEM). Among them, FIB-SEM alternately uses scanning electron imaging and focused ion beam milling of the top of the tissue block (Briggman and Bock, 2012). As a result, FIB-SEM can obtain in-situ images with a higher axial resolution to avoid complicated serial image registration and image defects. However, it has several drawbacks, including high cost, slow imaging speed, and sample destruction. As another methodology, ssEM has been used in many large-volume reconstruction projects (Hildebrand et al., 2017; Zheng et al., 2018; Macrina et al., 2021; Shapson-Coe et al., 2021) in recent years. ssEM is a suitable technique for large-volume reconstruction because it can image sections in parallel and has a large field of view. It cuts tissue into ultrathin serial sections and images them (Briggman and Bock, 2012) *via* electron microscopy. Then, those ssEM images are registered and overlaid to form a three-dimensional image stack for the following analysis. Nevertheless, ssEM contains many weaknesses, including section damage, misalignment, and poor axial resolution.

Section damage is caused by numerous factors during section preparation. The drying and dehydration of the section and the fixation of the tissue at different temperatures will cause section shrinkage (Fox et al., 1985; DurgunYucel et al., 1996; Gardella et al., 2003). In addition, the knife blade cuts through the tissue block, which causes the compression of the tissue along the *z*-axis (Gardella et al., 2003). Besides, due to ultra-thin section thickness, the cutting process may lead to shear deformation, tearing, and even loss of the sections (Dauguet et al., 2007; Agarwal et al., 2018). Moreover, staining and mounting can also cause severe tissue damage, such as cracking or folding (Choe et al., 2011), which cannot be avoided even if these sections are prepared by a section specialist (Popovych et al., 2020).

**FIGURE 1:**
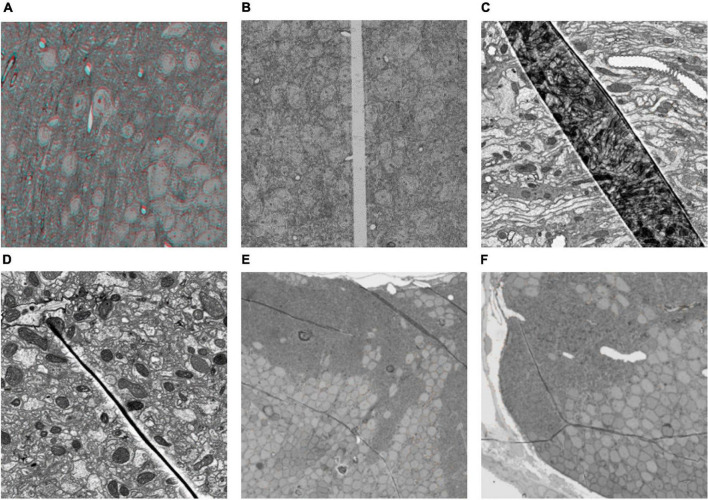
Some typical section damage images imaged by scanning electron microscope. **(A)** Continuous deformation. The entire section has been registered to the reference section, but there is still some local deformation. This picture shows the superposition of the red channel of the previous section and the blue and green channels of the next section. The presence of ghosting means that the previous and next sections are not registered perfectly. **(B–F)** Different types of broken sections.

Section damage in ssEM images has a negative influence on the subsequent segmentation. Severe section damage, such as cracks and folds, will lead to information loss and significant deformation. Common types of section damage are shown in Figure 1. Figure 1A shows the ssEM image with continuous deformation. Under the influence of internal and external forces during the sectioning process, the section generates global and local continuous deformation. In contrast to continuous deformation, discontinuous deformation is caused by cracks or folds. The continuity of these two kinds of deformation is broken at the crack or fold location. The tissue on both sides of the fold moves toward the fold (Figures 1C–F), while tissue on both sides of a crack moves away from the crack, as shown in Figure 1B. As shown in Figure 1, the crack or fold present great individual differences in appearance. The crack is relatively simple and causes less information loss and deformation (Figure 1B), while the fold is much more complex. Figure 1C is a deep fold with large deformation and information loss. Compared to the deep fold, shallow folding, as shown in Figures 1D–F, has smaller deformation and information loss, but the direction and degree of deformation may continue to change across the section. To make matters worse, these different types of cracks and folds can exist simultaneously on the same section, which results in the section with this damage not being easily repaired even by humans. Thus, it is necessary to develop an algorithm to address these severe damages.

Many works have been proposed to address the broken sections. Berlanga et al. (2011) proposed that thicker sections can avoid tissue tears. However, increasing the section thickness decreases the longitudinal resolution of the reconstructed volume. As a simple disposal method, some works (Yushkevich et al., 2006; Lein et al., 2007) removed the damaged sections to alleviate the complexity of the subsequent serial section registration. While section thickness is usually set to 30 nm in connectomics studies and the axon can be less than 100 nm in diameter (Popovych et al., 2020), too many removed sections may result in the difficulty of neurite tracing across sections. As a result, the registration of the damaged section is a worthwhile study for restoring the underlying neuronal structure as much as possible, especially in large connectomics projects.

The registration of the damaged section is not referred specifically to in commonly used serial section registration methods (Saalfeld et al., 2012; Yoo et al., 2017; Kajihara et al., 2019). These methods mainly focus on how to model the continuous nonlinear deformation of the unbroken section, such as the elastic model (Saalfeld et al., 2012), the convolutional neural network (CNN) model in ssEMnet (Yoo et al., 2017), and the blending of several rigid transformations (Kajihara et al., 2019). To improve the robustness of the registration results, the smoothness constraint of the deformation field is often appended, which has achieved good results on unbroken sections. However, it is helpless against the discontinuous deformation in the broken sections shown in Figures 1B–F.

To solve the registration problem of discontinuous deformation caused by cracks or folds, Pitiot et al. (2006) clustered a deformation vector to divide the damaged section into subregions and registered the subregion separately. Caesar (Popovych et al., 2020) used a CNN to segment the damaged section. SEAMLeSS (Mitchell et al., 2019; Macrina et al., 2021) broke the smoothness constraint at cracks or folds to simulate discontinuous deformation. Huang et al. (2020) focused on sections with folds, which were divided into two parts, and a CNN was used to generate the deformation field and restore the intermediate layer content. These methods divided the sections into completely separated areas and cannot address the shallow fold well in Figures 1D,E.

This paper proposes a method to register the damaged section. It calculates the expected affine of each coordinate point of the section. Unlike (Kajihara et al., 2019), the generated deformation field can model the discontinuous deformation caused by cracks, folds and nonlinear continuous deformations. Unlike other registration methods for specific types of damaged sections, this method is suitable for the registration of most damaged sections.

The key contributions of this paper are as follows:

• It proposes a registration method for damaged sections, which contributes to improving the accuracy of the reconstructed volume of biological tissues in connectomics studies.
• A new strategy is proposed for image matching. It uses a local model to select matching pairs from images to be matched, overcoming the disadvantage of the global model, which cannot identify matching pairs in areas with large deformation.
• The path distance is used to model the relationship between points on the section, so the generated deformation field can approximate not only continuous deformation but also discontinuous deformation.

## Materials and methods

**FIGURE 2:**
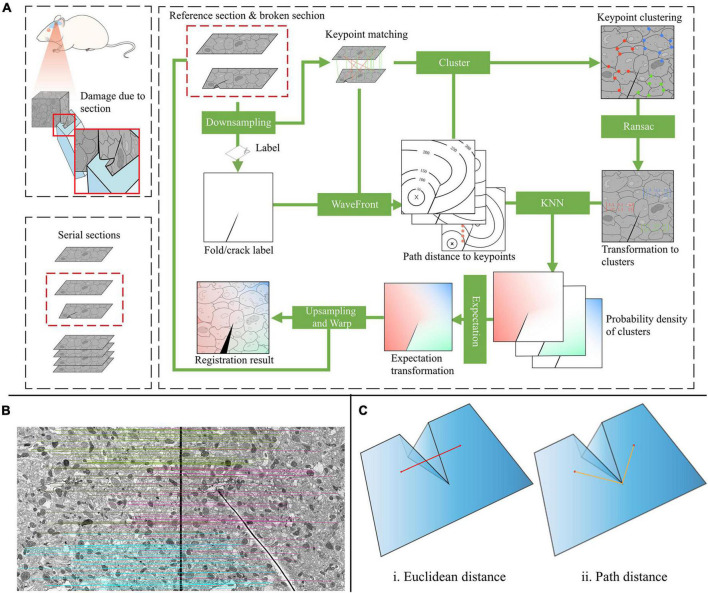
**(A)** Appearance damaged section and the overall excepted affine pipeline. **(B)** The match between the reference section and the folded section. Different colors represent different clusters. **(C)** Correlation measures between points on different sides of the fold.

Registering damaged sections presents numerous challenges that are difficult to overcome with common serial section registration methods. Here, we propose a novel method for registering damaged sections. The main step of the proposed method is depicted in Figure 2A. A CNN approach is utilized to extract corresponding points in adjacent sections (Section “Feature extraction and matching”). Then, a novel matching strategy is proposed to determine the complicated deformation within damaged sections, which is described as multiple local transformations (Section “Local transform estimation”). Finally, to generate the final deformation field, the expected transformation at each position is calculated with these local transformations (Section “Expected affine calculation”), and to combine continuous and discontinuous deformations, path distance is used to model the relationships between points on the section (Section “Probability density estimation”).

### Feature extraction and matching

Registering damaged sections requires a sufficient number of uniformly distributed matching pairs to characterize the correspondence between sections. CNNs have made great achievements in feature extraction. Therefore, the pretrained CNN model named SuperPoint (DeTone et al., 2018) is used to extract key points and descriptors. The SuperPoint model consists of an encoder and two decoders. The model input a W × H grayscale image and output a W × H heatmap and a W × H × 256 descriptor tensor. Then the key points are converted from the heatmap, and the descriptors are generated by interpolating the tensor. The pretrained model was trained in MS-COCO 2014 (Lin et al., 2014). It has good generalization performance. Therefore, this method is used without fine-tune. The corresponding descriptors are matched *via* the nearest neighbor matcher, and the match pairs are selected by the method descripted in Section “Local transform estimation.”

### Local transform estimation

A single global transformation cannot model the deformation of the registration of damaged sections. As shown in Figure 2B, these are two consecutive sections in the serial, and the section on the right is folded. The transformations on both sides of the fold can be approximated by two different affine matrices. Moreover, the affine matrices should be smoothly transitioned to ensure registration result continuity.

This paper uses K-means to automatically divide the matching pairs obtained in the previous step into different clusters and estimate the affine matrix in each cluster. Key point coordinate *kp*_*moving*_ of the moving image *I*_*moving*_ is used as the feature vector *V_f_* for clustering. K-means is used to cluster matching pairs into $\hat{k}$ clusters in the feature space of *V_f_*. In clustering, the cracks or folds are labeled [in this paper, we labeled those cracks or folds manually, but they can also be labeled by a CNN (Macrina et al., 2021)], and the path distance bypassing the cracks or folds is used to measure the similarity between clusters. The key points with similar spatial positions are clustered into the same cluster. In contrast, the key points on different sides of the fold are clustered into different clusters. Therefore, the affine matrix can describe the local transformation more accurately. Random Sample Consensus (RANSAC) (Fischler and Bolles, 1981) is used for each cluster *c_i_* to reject the incorrect matching pairs and estimate the affine matrix *A_i_* for each cluster. Clusters with too few inliers are discarded. After that, *r* clusters *c*_1_,*c*_2_,…,*c*_*r*_ and their corresponding key point sets and affine matrix sets are obtained.

### Expected affine calculation

In the previous step, the affine matrix of local key points was obtained. To complete the registration, it is necessary to know the transformation matrix of each coordinate point. Considering key points as sampling points in image space, the probability density of each cluster of *c_i_* is calculated by KNN (Hart et al., 2000) as


(1) $$p_{n}\,\left(x,c_{i}\right)=\frac{k_{i}/n}{V_{x}}$$

where *x* is the coordinate of each pixel, *n* is the total number of sample points, and *V_x_* is the volume including the *k*_*i–th*_ nearest sample point in *c_i_*. Then, the posterior probability of each coordinate point *x* belonging to *c_i_* is


(2) $$P_{n}\left(c_{i}|x\right)=\frac{p_{n}\,\left(x,c_{i}\right)}{\sum_{j=1}^{r}p_{n}\,\left(x,c_{j}\right)}$$

Finally, the expected affine matrix of each coordinate point can be calculated as follows:


(3) $$E_{n}\left(A_{x}\right)=P_{n}\left(c_{i}|x\right)A_{i}$$

### Probability density estimation

The accuracy of the estimated probability density $p_{n}\,\left(x,c_{i}\right)=\frac{k_{i}/n}{V_{x}}$affects the accuracy of the final expected affine matrices *E*_*n*_(*A*_*x*_). In most cases, *V*_*x*_=*d*^2^, *d* is the Euclidean distance between *x* and the *k*_*i–th*_ nearest sampling point. However, as can be seen in the left half of Figure 2C, in our case, the correlation between two red points can be broken by cracks or folds. Therefore, using Euclidean distance *d* to calculate probability density is not reasonable.

Sometimes the crack or fold does not go completely across the entire section, so the two points are not completely uncorrelated. Therefore, we use the path distance bypassing the crack or fold as shown in the right half of Figure 2C to measure the correlation between two points. To estimate the probability density, the distance from all pixels to the sampling point should be calculated. Compared with the popular path planning algorithms (Floyd, 1962; Hart et al., 1968), which obtain the path distance between two points, the wavefront expansion algorithm (Barraquand et al., 1992) can obtain the path distance between all pixels and the sampling point at one time without saving the path, so it has lower time complexity. Thus, the wavefront expansion algorithm is used to calculate the path distance.

The path distance is used to model the *d* in *V_x_*. Then, the probability density can be computed by Eq. 1. As a result, the generated deformation field can simulate not only continuous deformation but also discontinuous deformation.

### Implementation details

In the SuperPoint, the key points are detected above the confidence threshold at 0.015. In serial section registration, the distribution of the extracted key points on the section is generally not uniform. To obtain evenly distributed key points, we divide sections into patches to extract the key points. In this paper, the section is divided into 10 × 10 patches. Each patch extracts up to 50 key points according to the heatmap value to force the key points to be evenly distributed. In K-means clustering, *k* = 20. In calculating the probability density using KNN, *K* = 3.

## Results

We evaluate three aspects of expected affine (EA):

• The matching result between EA and RANSAC.
• The performance of registering typical damaged sections.
• The 3D reconstruction result with/without EA.

We evaluated the results of our method on several representative damage sections. These sections include all the damage types mentioned in Figure 1. Due to time and resource consumption considerations, we scaled the sections down. In the experiment, the cracks and folds were labeled manually.

The sections used in the experiment are available at https://github.com/TongXin-CASIA/Damaged_Section. Before the experiment, we performed histogram equalization on all sections.

### Matching performance

Matching key points is an essential part of the registration algorithm. The performance of the corresponding relationship directly affects the accuracy of the registration algorithm.

The purpose of matching in the EA algorithm is to obtain more evenly distributed matching pairs in damaged sections, which is a characteristic that has not been considered in most matching methods. In this experiment, EA is compared with RANSAC, which also has the ability of selecting matching pairs. The matching effect is evaluated on real damaged sections.

**FIGURE 3:**
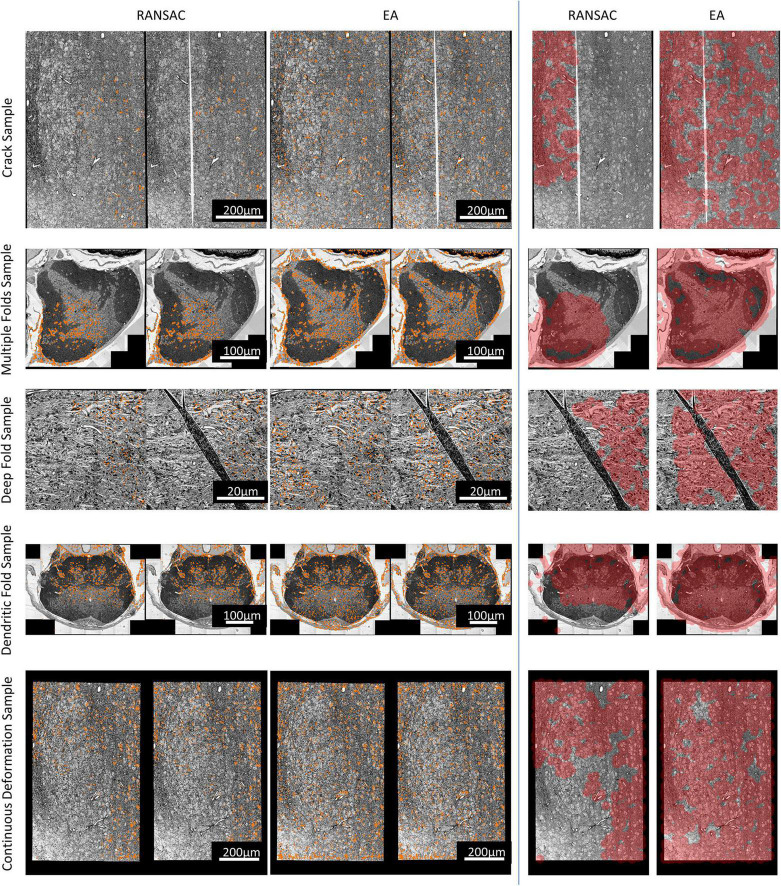
The match results. Left: The matched points in the sections. For each pair, the left is the reference section, and the right is the damaged section to be registered. Right: Area covered by correspondences on different sections (the shorter side of each section is scaled to 1,000 pixels, and the red-colored area represents the area covered by correspondences).

Figure 3 shows the result of feature matching. When the distance between a matching pair is less than three pixels, it is considered to be a correct match. The input of EA and RANSAC is the same.

The number and rate of inliers shown in Table 1 represent the efficiency of utilizing matched pairs, and more matching pairs indicate better matching quality, which results in better registration accuracy. It can be seen that our method has more matched pairs and fewer outliers than RANSAC. In addition, as illustrated in Figure 3, our method produces more evenly distributed matching pairs than RANSAC, and this advantage can also be demonstrated by the Area% index in Table 1. Area% represents the proportion of the matching area on the section. We used a circle centered on each match point with a radius of 35 pixels to define the matching area (the shorter side of each image is scaled to 1,000 pixels). The area of all matching areas divided by the area of the section image is regarded as Area%. A larger value of Area% indicates that the matching algorithm can consider a more global transformation of the damaged section. The right part of Figure 3 shows that the correspondences obtained by our method cover a larger area.

**TABLE 1:** Quantitative analysis of matching results.

Sections	Method	Time (s)	Match num	Inliers num	Inliers%	Area%
Crack sample	RANSAC	0.467	2147	170	7.9	17.4
	EA	139.836		**783**	**36.5**	**68.4**
Multiple folds sample	RANSAC	0.346	2207	587	26.6	32.2
	EA	50.789		**1448**	**65.6**	**75.4**
Deep fold sample	RANSAC	0.389	2020	211	10.4	31.4
	EA	9.988		**589**	**29.2**	**62.6**
Dendritic fold sample	RANSAC	0.350	2654	1179	44.4	51.5
	EA	58.254		**1906**	**71.8**	**75.9**
Continuous deformation sample	RANSAC	0.479	2389	759	31.8	46.5
	EA	337.637		**1332**	**55.8**	**73.9**

The best results are highlighted in bold.

In conclusion, our method is significantly better than the RANSAC algorithm in terms of the number of matching pairs, the proportion of inliers, and the universality of the distribution of matching pairs. Furthermore, from the comparison of the experimental results, it is clear that our method produces better results when there are large cracks or folds in the sections.

### Registration performance

In the last section, we compared the matching effects of RANSAC and our method in damaged sections. Experimental results show that the effect of our method is much better than RANSAC. In this section, we demonstrate the registration performance of our method quantitatively and qualitatively.

**FIGURE 4:**
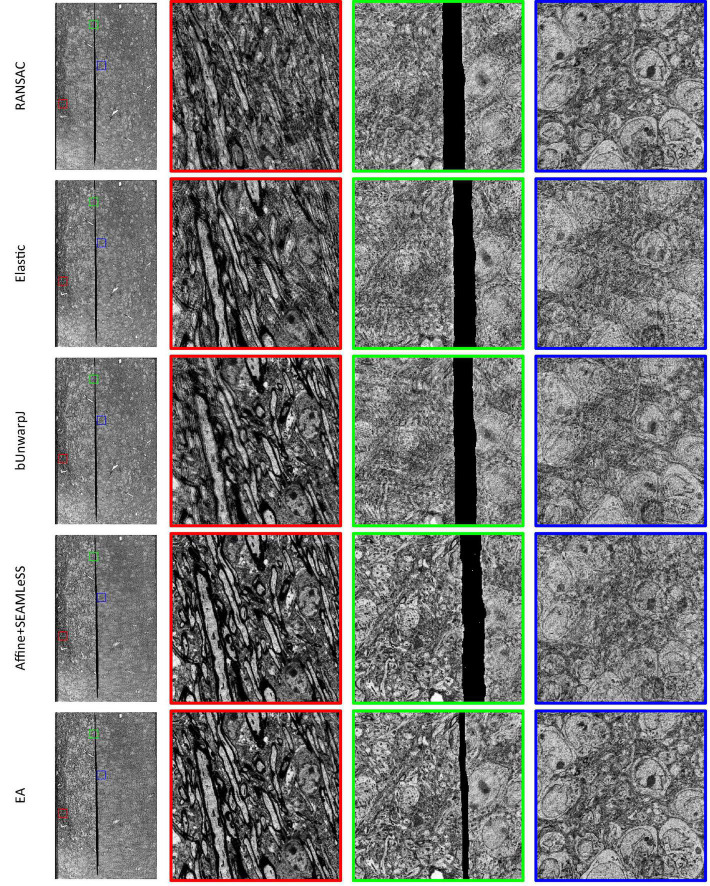
The registration results of Crack Sample, which were generated by superimposing damaged sections and reference sections. A location with noticeable ghosting indicates that it is not registered well. The complete registration results can be obtained from the Supplementary Materials.

**FIGURE 5:**
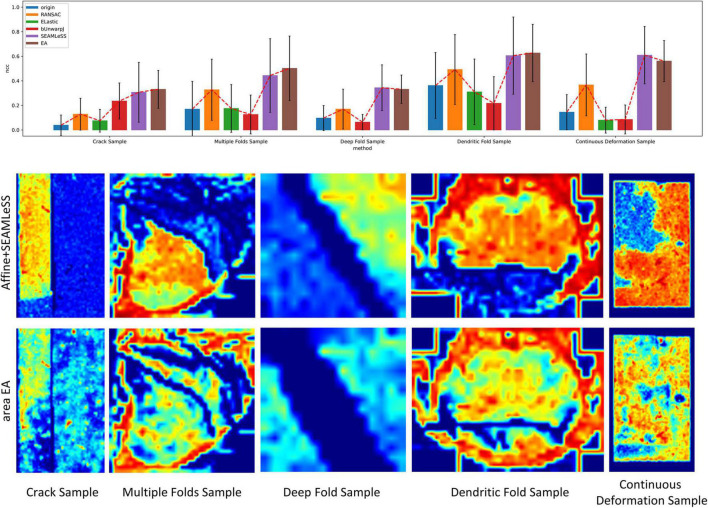
The local patch normalized cross-correlation (NCC) of registration results. Up: Box plot of the mean of the local patch NCC distribution. Bottom: Heatmap of the NCC. The red indicates a high degree of similarity between the registered section and the reference section at the current position, whereas the blue indicates the inverse.

Expected affine, RANSAC, Elastic (Saalfeld et al., 2012), bUnwarpJ (Arganda-Carreras et al., 2006), and SEAMLeSS (Mitchell et al., 2019; Macrina et al., 2021) are used to estimate the transformation of damaged sections. Because SEAMLeSS is a fine registration method, we use an affine transformation to perform coarse registration on the sections beforehand. In addition, SEAMLeSS and EA use the same cracks or folds labels obtained manually, while the other methods do not require the cracks or folds label. According to the calculated transformation, the damaged section is warped and repaired. The qualitative results, depicted in Figure 4, are obtained by overlaying the reference and the registered results of damaged sections. Due to space limitations, we show only the registration results for the Crack Sample here. The complete results are available in the Supplementary materials. We also analyze the registration results quantitatively. Due to the nonlinear deformation of the damaged section, we examine the registration result locally through the whole section. As a result, we divide the registered images into 64 × 64 patches and evaluate the registration accuracy of each patch using normalized cross-correlation (NCC). As plotted in the up part of Figure 5, our method has good results in all sections. Although it is difficult to distinguish SEAMLeSS and our method in terms of the mean value of the NCC of local patches, our method has a much smaller standard deviation. Furthermore, we illustrate the heatmaps of the NCC for SEAMLeSS and the proposed method in the bottom part of Figure 5. This figure can show the registration accuracy of our method in different locations.

#### Results of continuous deformation

The Continuous Deformation Sample is an entire section with large continuous deformation. The bottom part of Figure 5 shows that the red (higher-precision) covers a larger area in the proposed method results. This result means that the proposed method can deal with continuous deformation well.

#### Results of discontinuous deformation

All other samples contain discontinuous deformation. As shown in Figure 4, ghosting is clearly visible for all the methods except for EA for Crack Sample. The heatmaps also show that for those samples containing discontinuous deformation, the SEAMLeSS, which achieves the best result of other methods, can only obtain a good result on one side of the crack or fold. Except for EA, other methods are inadequate for dealing with large discontinuous deformations caused by cracks or folds.

The results of continuous deformation and discontinuous deformation indicate that our method can be used to address both continuous and discontinuous deformation.

### 3D reconstruction

The volume assembly of ssEM is to register the ssEM images sequentially and then stack these images in the longitudinal direction. Damaged sections, especially broken sections, are a tremendous challenge for the 3D reconstruction of ssEM volume. Here, we compare the 3D reconstructed structure with and without the proposed method.

**FIGURE 6:**
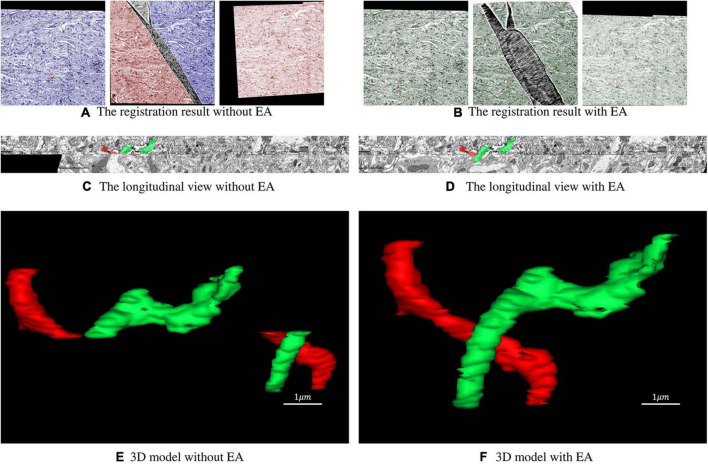
Registration result of serial sections. **(A)** The registration result of the folded section with its previous and subsequent sections without the expected affine. The blue part of the folded section represents the area corresponding to the section of the previous layer, and the red part represents the area corresponding to the section of the next layer. **(B)** The registration result of the folded section with its previous and subsequent sections with expected affine. The green area of the folded section corresponds to the previous and next sections. **(C,D)** Longitudinal view of the registration result. **(E,F)** 3D model reconstructed from the registration result.

The dataset used in this experiment was acquired from the optic lobe of Drosophila and imaged using SEM with a voxel resolution size of 3 nm × 3 nm × 50 nm. The dataset size is 10,000 × 10,000 × 64. The sections were scaled to 1,000 × 1,000 for the experiment. The 32nd layer in this dataset is a folded section where the fold goes across the whole sections shown in Figure 1A. The proposed method is used to register the damaged section to the reference section. After that, these sections are registered sequentially and stacked into a 3D volume. Two neurites are labeled in the volume. Figures 6A,C,E illustrates the registration result of original serial sections that are not repaired, and Figures 6B,D,F illustrates the registration results for serial sections repaired with EA.

As shown in Figures 6A,B, the continuity of the transformation between the previous and the next sections of the folded section is preserved with EA. To further evaluate the influence of the proposed method, we label two neurites with different colors. Figures 6C,D shows that without EA, the continuity of the serial section stack along the longitudinal direction is very poor, and the continuity is much improved with EA. The 3D view of the reconstructed neurites in Figures 6E,F also confirms the results.

## Discussion

Section damage is unavoidable during sample preparation in ssEM. Registering the damaged sections to the reference sections for repair is a suitable method. However, along with the more common continuous deformations, discontinuous deformations caused by cracks or folds may exist on the sections. It is difficult to register the damaged sections with common registration methods. To solve this challenge, we modeled these two deformations using the path distance bypassing the damaged area. The proposed method can resolve both continuous and discontinuous deformation in the section simultaneously.

The previous section of the damaged section is commonly used as the reference section. However, sometimes there will be discontinuous deformation in multiple consecutive sections. These sections are not suitable to be regarded as reference sections. Therefore, in practical application, the entire section closest to the damaged section in the serial section is used as the reference section for repair.

However, due to the high time complexity of path planning, the proposed method is significantly slow. To deal with this shortcoming, the damaged section can be scaled down properly, and the estimated deformation field is enlarged to warp the full-size section.

In this paper, the label of the cracks or folds is obtained manually, which is also time-consuming work for complex damaged sections. Automatically detecting and labeling folds and cracks are the direction of upcoming research. Furthermore, the recovery of the lost information in crack and fold areas is also worthy of investigation.

## Data availability statement

The datasets presented in this study can be found in online repositories. The names of the repository/repositories and accession number(s) can be found below: https://github.com/TongXin-CASIA/Damaged_Section.

## Ethics statement

The animal study was reviewed and approved by Peking University and CAS Center for Excellence in Brain Science and Intelligence Technology.

## Author contributions

TX, XC, and HH contributed to conception and design of the study. TX implemented the algorithm and wrote the first draft of the manuscript. LS prepared the experimental environment and organized the database. LL completed sample preparation and imaging. All authors contributed to manuscript revision, read, and approved the submitted version.
