# Supplementary material for: Expected affine: A registration method for damaged section in serial sections electron microscopy
Source: Front Neuroinform. 2022 Sep 2;16:944050. doi: 10.3389/fninf.2022.944050 (PMC9478550; doi:10.3389/fninf.2022.944050)
Supplement: Supplementary file 1 [file Data_Sheet_1.PDF]

Supplementary Material

1 Registration Results

The registration results for all samples. Images were generated by superimposing damaged sections and reference sections. The location with noticeable ghosting means that it is not registered well. Fig. 1 is an overview of all the results, and Fig. 2- Fig. 6 show the results for different sections.

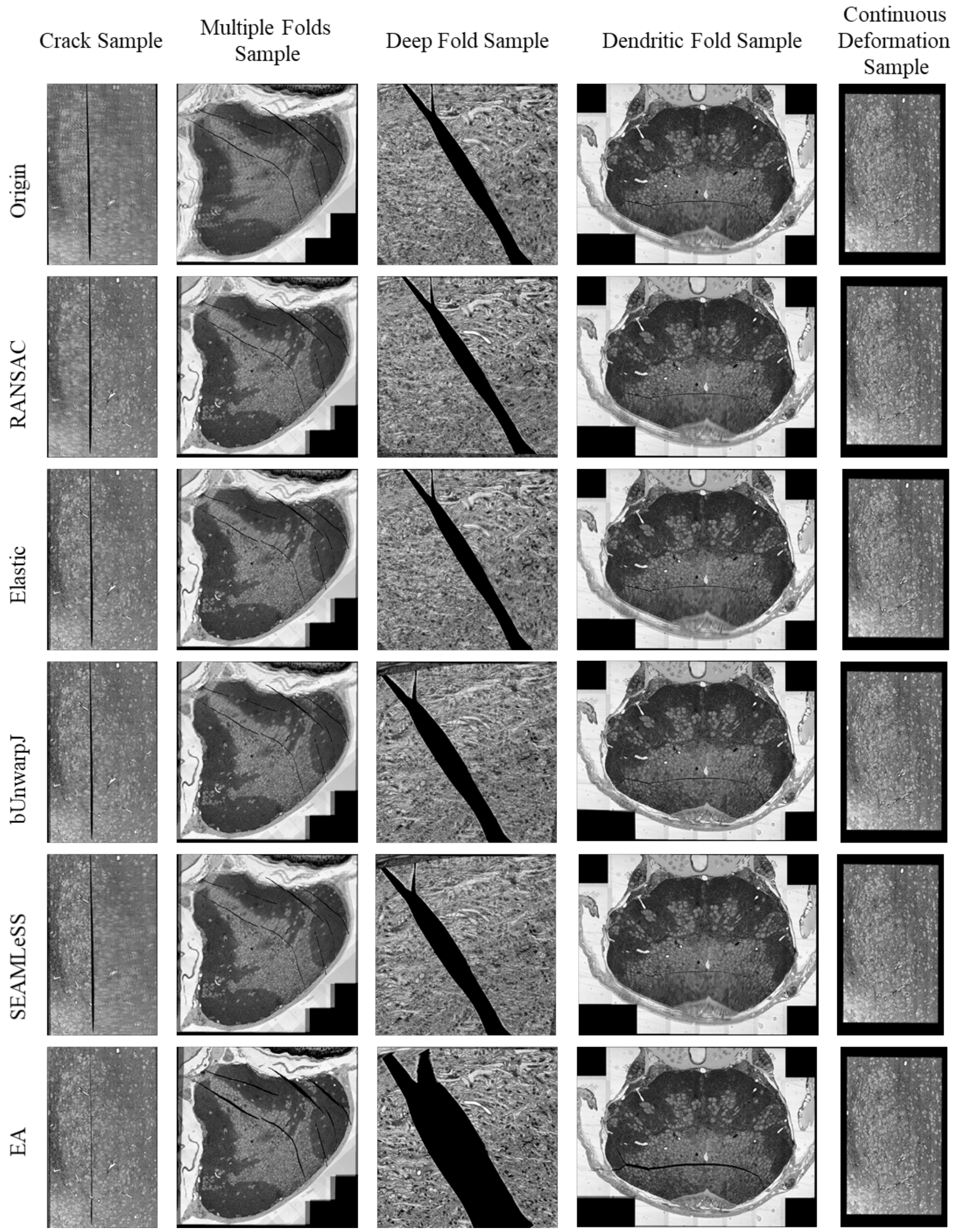

Supplementary Figure 1. Overview

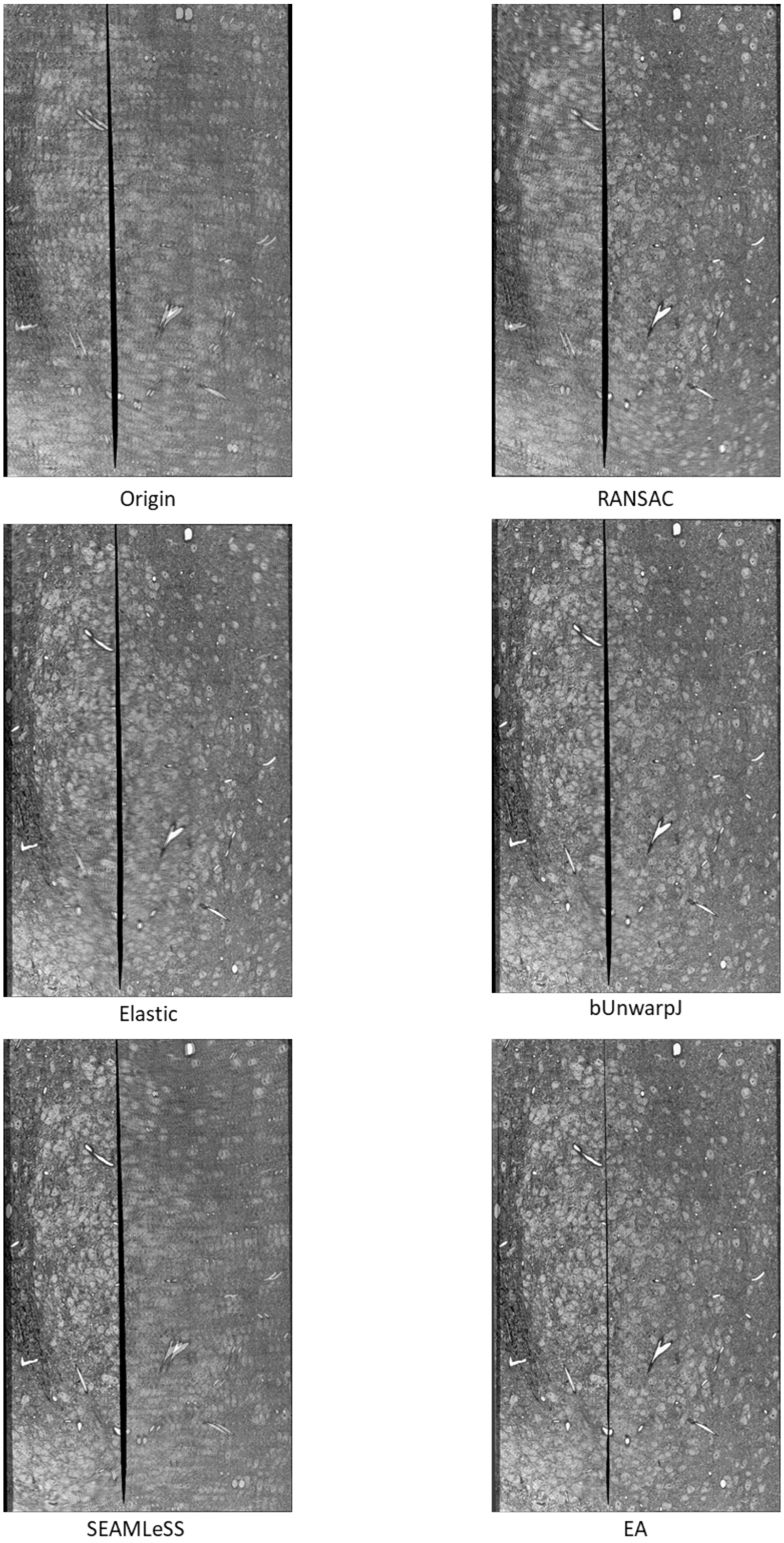

Supplementary Figure 2. Results for Crack Sample

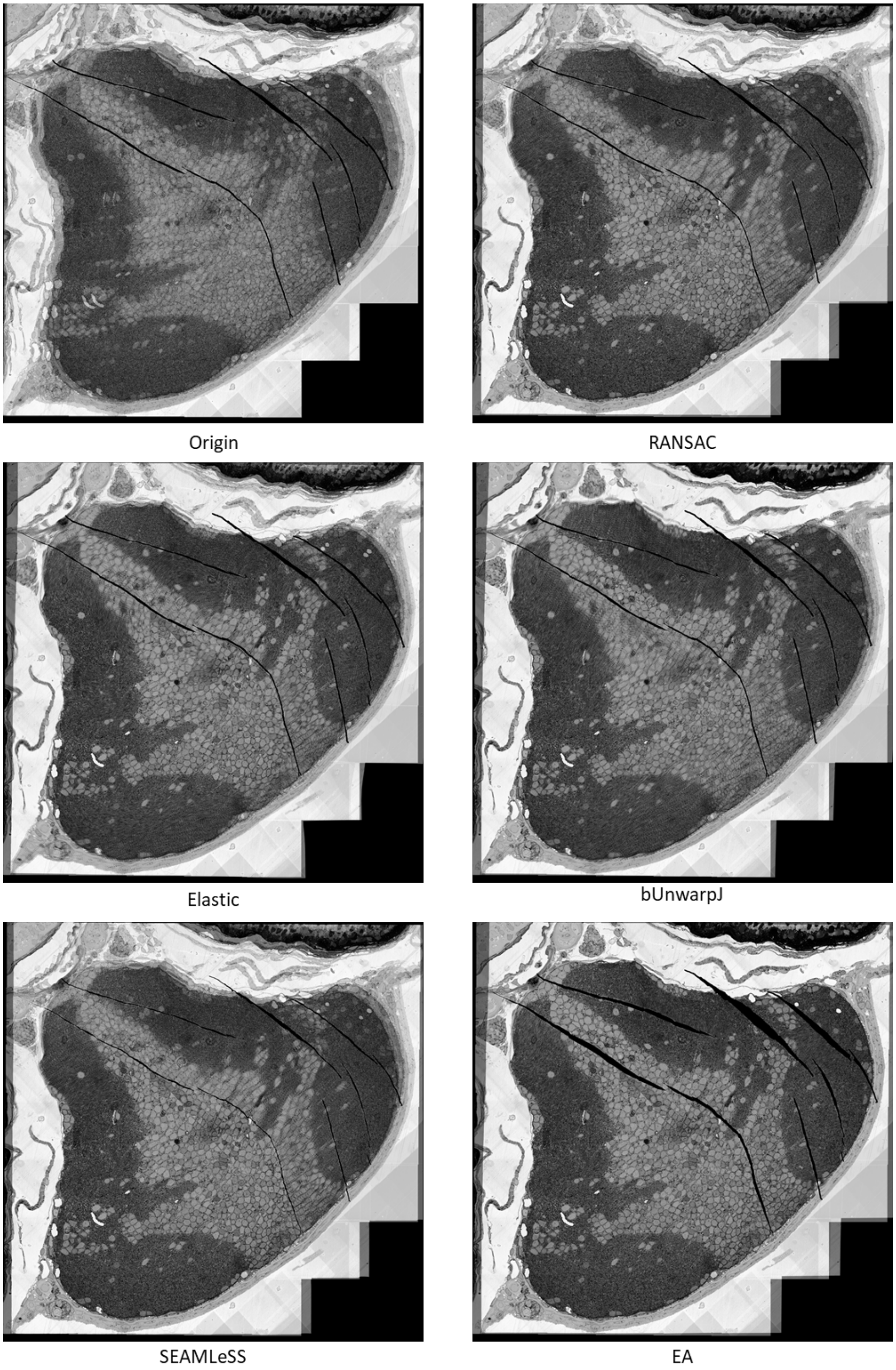

Supplementary Figure 3. Results for Multiple Folds Sample

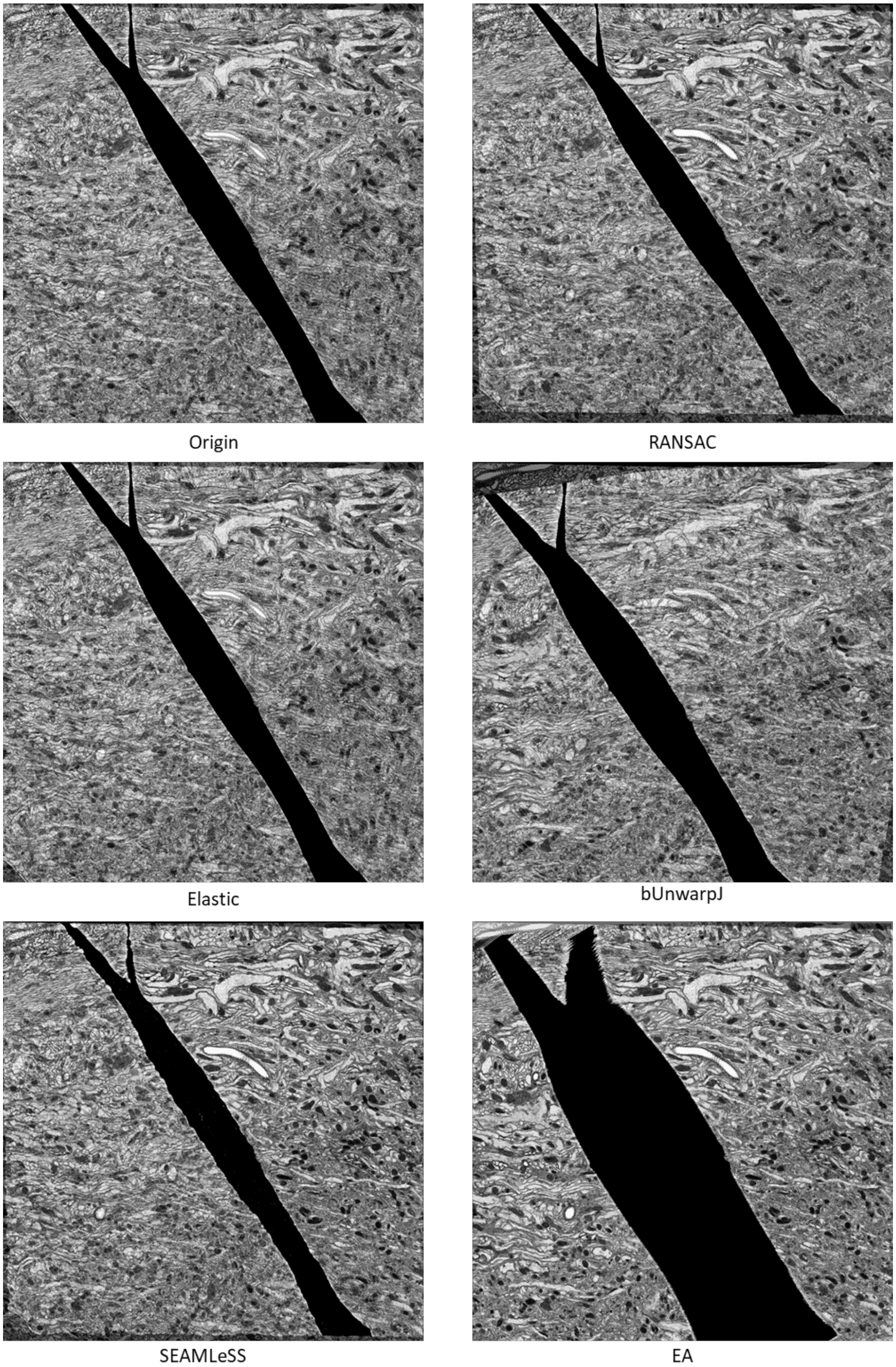

Supplementary Figure 4. Results for Deep Fold Sample

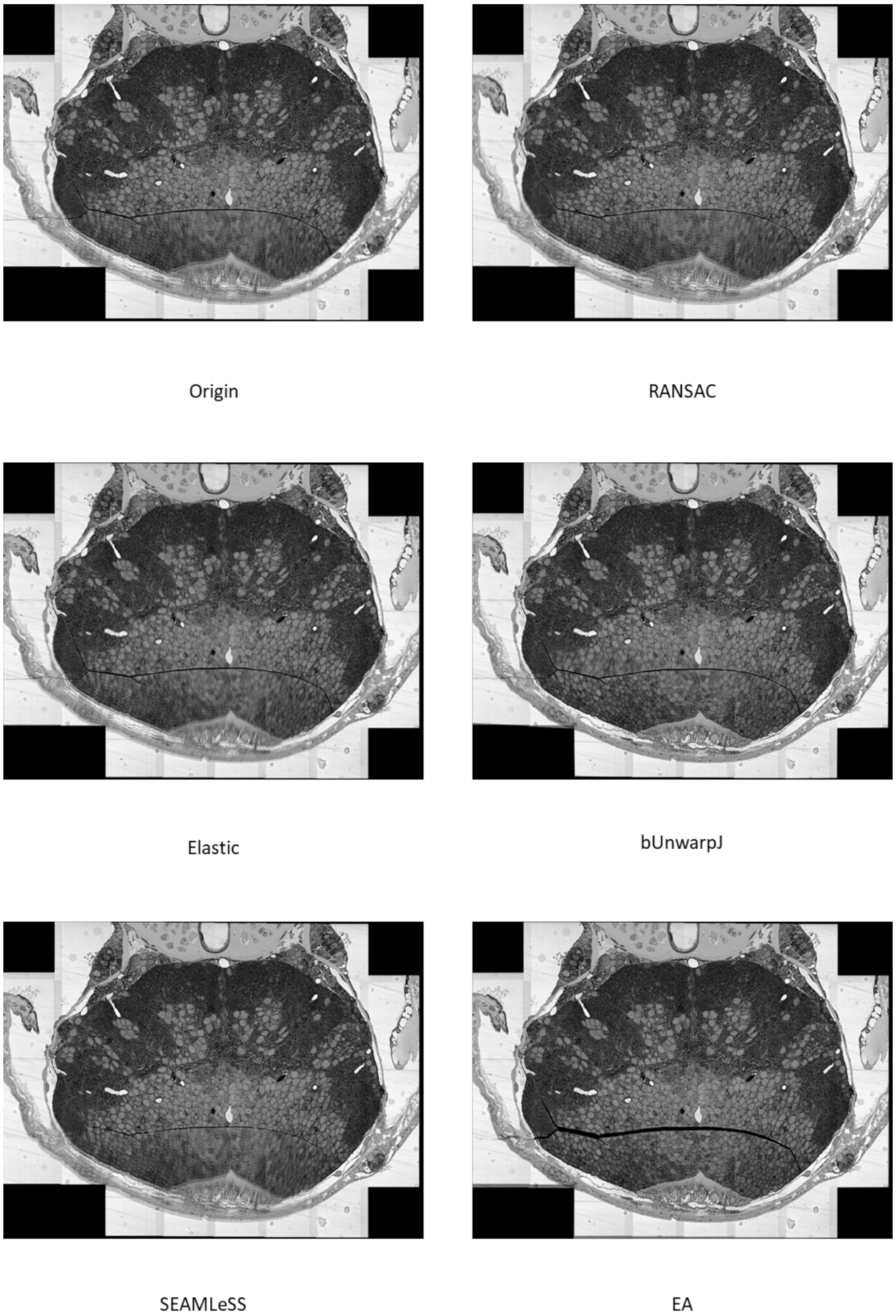

Supplementary Figure 5. Results for Dendritic Fold Sample

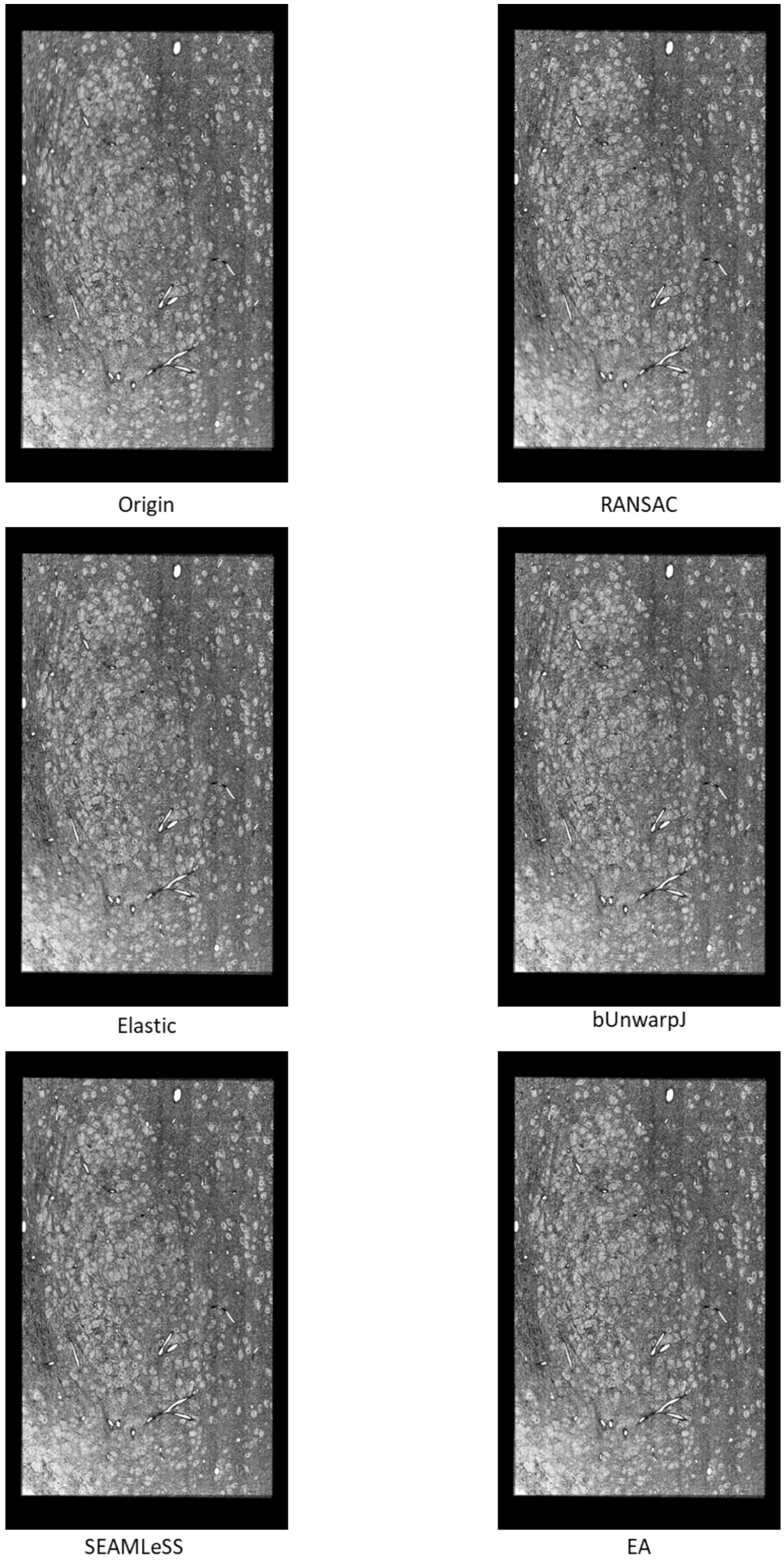

Supplementary Figure 6. Results for Continuous Deformation Sample

2 Local enlargement for Figure

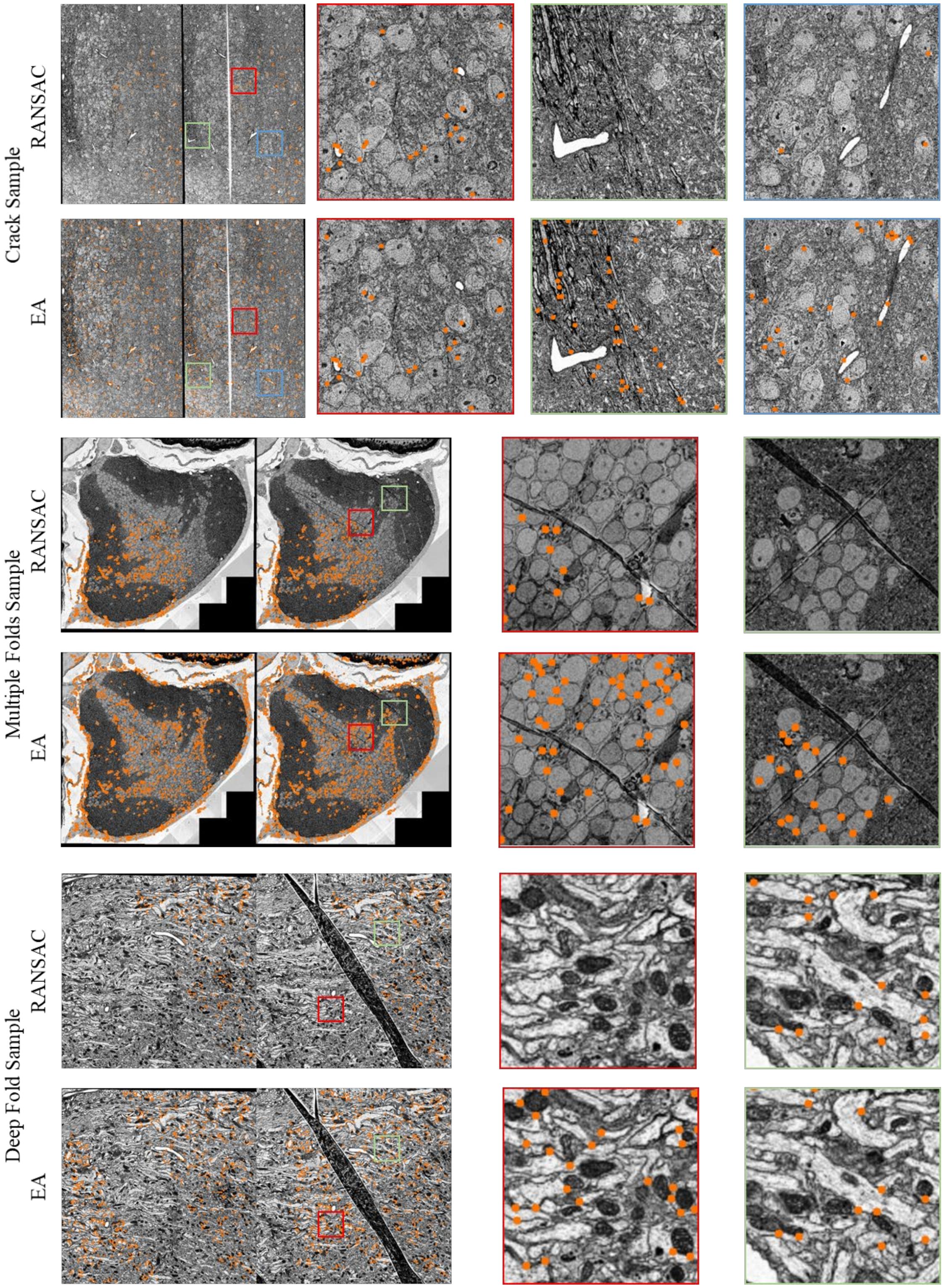

Supplementary Figure 7. Matching result for Crack Sample, Multiple Folds Sample and Deep Fold Sample.

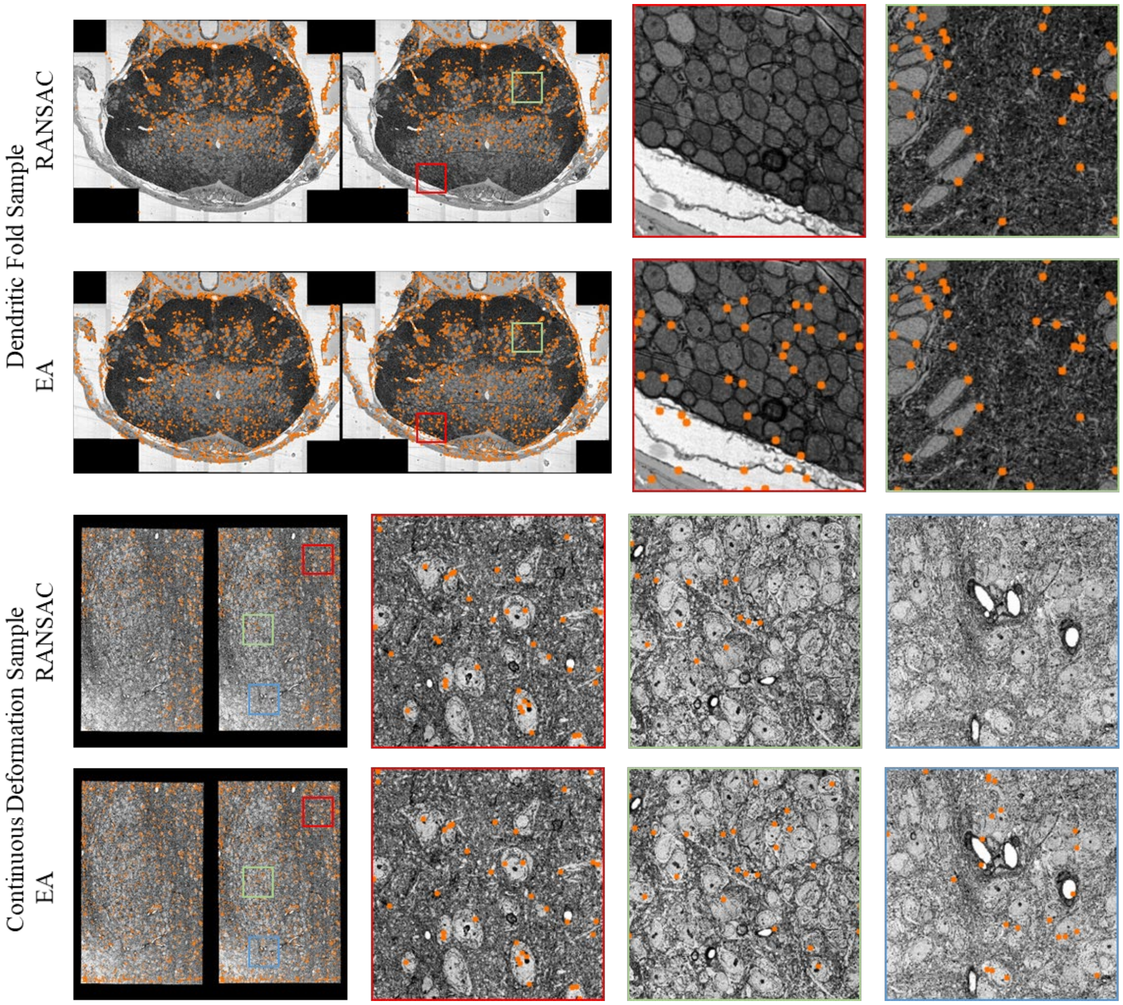

Supplementary Figure 8. Matching result for Dendritic Fold Sample and Continuous Deformation Sample.

3 Influence of different feature extractors on matching results

Enough matching pairs are needed to ensure the effect of registration. In this experiment, we quantitatively compare the matching results of SIFT and SuperPoint.

From Supplementary Table 1, we can see that SuperPoint achieves better matching results. On samples with good SIFT performance, such as Dendritic Fold Sample and Continuous Deformation Sample, SuperPoint can achieve similar results as SIFT. On the samples with poor SIFT performance, such as Crack Sample, Multiple Folds Sample and Deep Fold Sample. SuperPoint can achieve better results than SIFT. Especially in Deep Fold Sample, the deformation between sections is large, SIFT cannot obtain any effective correspondence, while SuperPoint can still obtain good results. In addition, the RANSAC and EA methods are random algorithms so the experimental results may be slightly different from the corresponding results in the manuscript. However, the conclusion is not affected.

Supplementary Table 1. Matching results between SuperPoint and SIFT(Lowe, 2004).

			RANSAC			EA		
Sections	Methods	Match num	Inliers num	Inliers%	Area%	Inliers num	Inliers%	Area%
Crack Sample	SIFT	1689	122	7.2	15.3	140	8.3	14.2
	SuperPoint	2139	172	8.0	15.4	786	36.7	68.4
Multiple Folds Sample	SIFT	2009	395	19.7	28.1	1097	54.6	67.7
	SuperPoint	2218	595	26.8	31.9	1436	64.7	74.7
Deep Fold Sample	SIFT	-	-	-	-	-	-	-
	SuperPoint	1902	183	9.6	29.0	550	28.9	61.6
Dendritic Fold Sample	SIFT	2287	1229	53.7	43.3	1640	71.7	61.0
	SuperPoint	2648	1178	44.5	51.7	1916	72.4	76.2
Continuous Deformation Sample	SIFT	2108	670	31.8	42.0	1227	58.2	70.4
	SuperPoint	2387	767	32.1	46.6	1321	55.3	74.4

4 Influence of different distance measures on registration results

This paper uses path distance instead of Euclidean distance to model discontinuous deformation. To verify the effectiveness of this measure, we use path distance and Euclidean distance to measure deformation correlation and compare the registration results.

The comparison results can be seen in Supplementary Figure 9. At the location far away from discontinuous deformation (red box), using the path distance and Euclidean distance achieve almost the same results. However, path distance can lead to better results in locations close to discontinuous deformation (other boxes). The results show that the path distance can better simulate the discontinuous deformation caused by folds or cracks than the Euclidean distance.

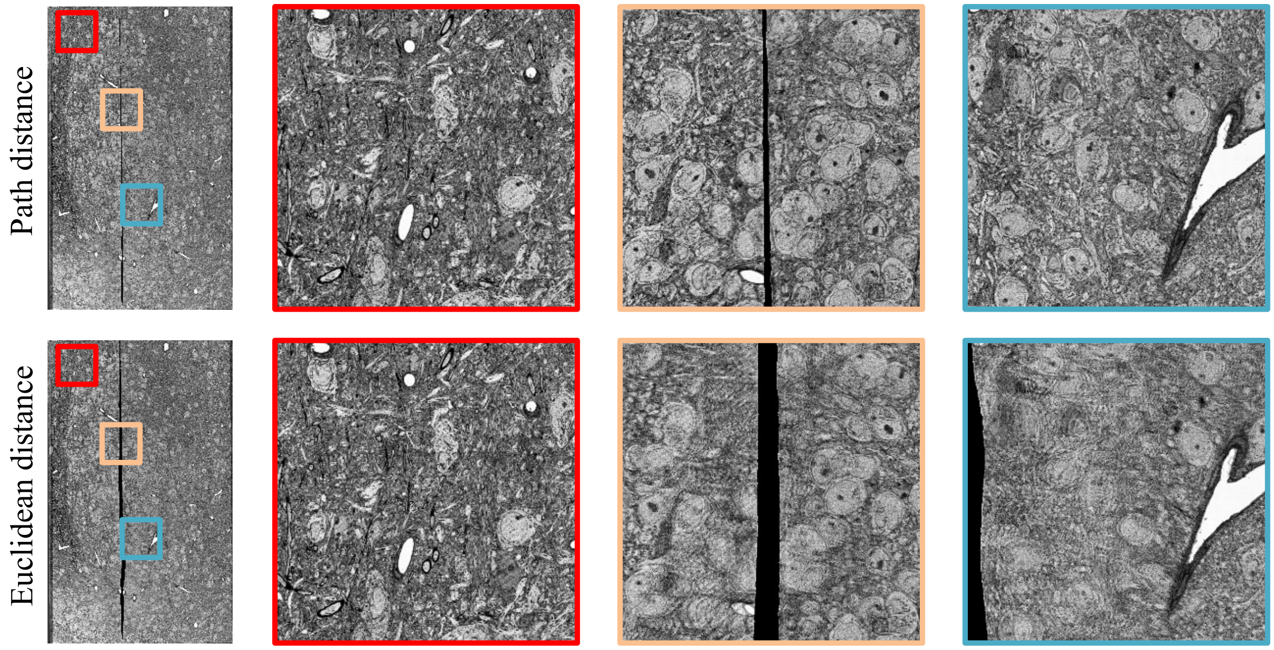

Supplementary Figure 9. The registration results of Crack Sample, which were generated by superimposing damaged sections and reference sections.

This conclusion can also be verified in Supplementary Figure 10. For samples with discontinuous deformation, using Euclidean distance leads to poor results near discontinuous deformation (folds or cracks). However, for the sample without discontinuous deformation, the results of using these two distance measures are almost identical. Therefore, we use path distance to model nonlinear deformation in this paper.

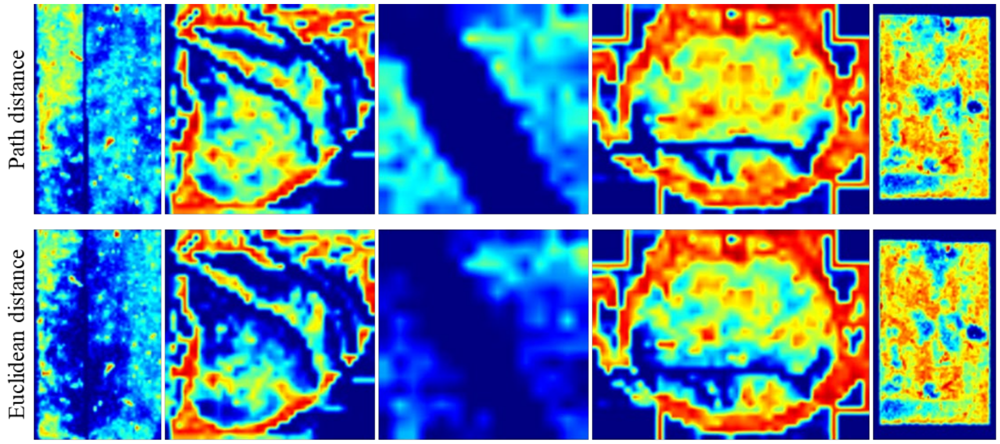

Supplementary Figure 10. Heatmap of the NCC. The red indicates a high degree of similarity between the registered section and the reference section at the current position, whereas the blue indicates the inverse.

5 Influence of experimental parameters on Area% of matching results.

Area% represents the proportion of the matching area on the section. The short side of the image is first scaled to a fixed size. In this case, the matching area is the area of the union of circles with a fixed radius centered on the matching point. In this paper, the short side is scaled to 1000 pixels, and the radius is set to 35 pixels. The influence of different values of these two parameters on Area% is shown in Supplementary Table 2.

Supplementary Table 2. Area% under different experimental parameters.

Sections		Crack Sample		Multiple Folds Sample		Deep Fold Sample		Dendritic Fold Sample		Continuous Deformation Sample	
Shorter Side	Radius	EA	RANSAC	EA	RANSAC	EA	RANSAC	EA	RANSAC	EA	RANSAC
500	20	77.1	17.2	78.6	33.6	66.0	31.9	79.0	54.4	79.6	50.8
	35	98.0	24.4	89.5	40.3	81.1	41.4	87.5	65.5	92.9	65.9
	50	100.0	29.8	92.2	45.0	88.9	47.0	91.4	73.6	96.9	74.3
1000	20	35.0	8.4	53.6	23.2	38.1	15.8	58.0	37.1	45.4	27.5
	35	68.4	15.4	74.6	31.9	61.6	29.0	76.3	51.7	74.4	46.6
	50	88.6	20.2	84.1	36.4	72.3	36.3	82.7	58.8	86.0	56.9
2000	20	11.4	2.8	26.9	11.4	14.7	5.3	29.1	17.8	17.0	10.1
	35	28.5	7.0	48.1	20.8	32.6	13.1	52.6	33.3	38.6	23.3
	50	47.1	11.1	62.7	27.0	48.0	21.0	66.5	43.5	57.5	35.1

6 Comparison of time consumption of different methods.

High time consumption is the biggest drawback of the proposed method. Here we compare the time consumption of different methods. This experiment was run on Intel (R) Xeon (R) gold 6142 CPU and NVIDIA Tesla V100. Except for the neural network method SEAMLeSS, which runs on GPU, all other methods run on CPU.

Supplementary Table 3. Time consumption of different methods.

Method	Crack Sample	Multiple Folds Sample	Deep Fold Sample	Dendritic Fold Sample	Continuous Deformation Sample
RANSAC	34.2s	13.8s	2.5s	16.3s	47.8s
bUnwarpJ	6.8s	4.7s	5.2s	8s	5.3s
Elastic	19.2s	10s	-	13.1s	11s
RANSAC+SEAMLeSS (GPU)	59.2s	23.4s	4.2s	26.8s	79.4s
EA	1417.4s	478.1s	70s	487.6s	2293.2s

Supplementary Reference

Lowe, D.G. (2004). Distinctive image features from scale-invariant keypoints. *International Journal of Computer Vision* 60(2), 91-110. doi: Doi 10.1023/B:Visi.0000029664.99615.94.
